# Photovoice and empowerment: evaluating the transformative potential of a participatory action research project

**DOI:** 10.1186/s12889-018-5335-7

**Published:** 2018-04-02

**Authors:** Kirsten Budig, Julia Diez, Paloma Conde, Marta Sastre, Mariano Hernán, Manuel Franco

**Affiliations:** 1 Social and Cardiovascular Epidemiology Research Group, School of Medicine, University of Alcala, 28871 Alcala de Henares, Madrid Spain; 2 Madrid Salud, Public Health Institute, Madrid City Council, Madrid, Spain; 3 Escuela Andaluza de Salud Pública, Biomedical Research Networking Centres (CIBER), Granada, Spain; 4 Biomedical Research Networking Center for Epidemiology and Public Health (CIBERESP), Madrid, Spain; 5 Department of Epidemiology, Johns Hopkins Bloomberg School of Public Health, Baltimore, MD USA

## Abstract

**Background:**

Photovoice is a visual research methodology with the intention to foster social change. Photovoice has been used to investigate change in empowerment in vulnerable communities, However, the individual experience of participants involved in Photovoice projects is seldom scrutinized. Our aim was to explore and describe the individual experiences of the female individuals who participated in a previous Photovoice project. We analyzed a change in the women’s empowerment in terms of: 1) gain in knowledge and skills, 2) change in self-perception, and 3) access to and use of resources.

**Methods:**

This qualitative study took place in the low-income District of Villaverde (Madrid, Spain), from January-June 2016. We conducted 10 semi-structured interviews with the female residents who had participated in the previous Photovoice project. We also collected field notes. We analyzed these data through a direct qualitative content analysis. The three outlined dimensions of empowerment provided guidance for the analysis of the results.

**Results:**

We found positive changes in the three dimensions of empowerment: 1) participants acquired new knowledge and developed critical awareness of their community; 2) the social recognition participants received transformed their self-perception; and 3) the project allowed them to expand their social networks and to build new links with different actors (research partners, local decision makers, media and the wider public).

**Conclusions:**

Photovoice projects entail the opportunity for empowering participants. Future research using Photovoice should assess the influence it has on participants’ empowerment changes and how to sustain these individual and social changes.

## Background

Photovoice is a visual research methodology that puts cameras into the participants’ hands to help them to document, reflect upon, and communicate issues of concern, while stimulating social change [1, 2]. With the intention to foster social change, Photovoice can enhance community engagement, increase awareness of community resources, and foster self-efficacy of the research partners [3]. Caroline Wang and Mary Ann Burris developed Photovoice, with the end goals of 1) to enable people to record and reflect their community’s strengths and concerns, 2) to promote critical dialogue and knowledge about important issues through small group discussions of photographs, and 3) to reach policy makers [1]. These are central elements of empowerment, a concept that is intertwined with the Photovoice methodology from its original conceptual underpinnings to its implementation.

Firstly, Photovoice builds on Freire’s methods of empowerment education [4] with one main element being the acquisition of knowledge that the participants collectively produce by reflecting on and discussing community issues. Secondly, Photovoice is able to ‘voice’ and represent individual perceptions [5]: this is an essential element of the Photovoice process and is often associated with empowerment, particularly emphasized in discourses on women’s empowerment [6]. Thirdly, feminist theory, is meant to empower vulnerable populations, and recognize local expertise that cannot be fully realized from the outside [7].

However, there is no universal definition of empowerment, and it is mostly used as a multi-faceted concept, adapted to the context it is being used in [8]. More recent conceptualizations, for example, relate it to a change in self-perception, in terms of the perceived control in different areas of life [9]. Such transformation has been described as a main empowerment outcome in previous studies using Photovoice [10–12].

Moreover, empowerment can encompass a change in how participants engage with their surroundings, that is to say their access to and use of resources and the formation and potential of social relations and networks. This concept has previously been used to investigate change in empowerment in vulnerable communities, such as populations living with HIV/AIDS [13], mothers with learning difficulties [14], or indigenous communities [7], among others. Despite the potential effects on individuals, especially those from vulnerable populations, few authors have particularly focused on what a Photovoice project actually means for the participants [10, 15, 16]. Evaluating this potential can increase our understanding of empowerment processes and generate valuable implications for future research using Photovoice leading to place-based social change projects [17].

The purpose of this study was to explore and describe the individual experiences of the female individuals who participated in a previous Photovoice project. Three dimensions served as points of reference for analyzing a change in the women’s empowerment: 1) gain in knowledge and skills, 2) change in self-perception, and 3) access to and use of resources. We chose these dimensions because these were most likely to be impacted by the involvement in a Photovoice project. Moreover, these dimensions reflect concepts from recent literature and are broad enough to include a range of different notions of these concepts. A change in self-perception, for example, may have an impact on self-esteem, another important marker for individual empowerment [16]. They are therefore not to be seen as strict categories but rather as points of orientation to guide this study.

## Methods

### Study context

Participants of this study were members who had participated in a previous Photovoice project [18, 19]. We carried out this previous project, branded as ‘Photovoice Villaverde’, as a collaboration with the Public Health Institute of Madrid within the Heart Healthy Hoods project (hhhproject.eu) [20, 21]. In the Photovoice Villaverde project participants used their photographs to describe and reflect upon their local food environment (for a short video on the project please see: https://youtu.be/VIiFggKzVas). The Photovoice Villaverde project and its results have been published elsewhere [18, 19].

The target study area for this project was the low-income District of Villaverde, located in the southeastern part of the city of Madrid, Spain. Two neighborhoods were selected within this District for the project. We used a purposive sampling strategy to engage participants, and based their recruitment on residence location. The resulting sample consisted of 24 participants (n=14 female participants).

Following Wang’s methodology [1], we divided them into small discussion groups, which met for (at least) five discussion sessions. In brief, we asked participants to *‘take pictures of all the features related to the food environment in your neighborhood over the next week’* in Session 1, in which a photographer provided digital cameras, and a photography workshop to participants. Sessions 2–4 consisted of small group discussion sessions, where participants reviewed their photographs and discussed them with the other group members. Participants themselves codified the data and identified the themes that emerged from these data (the photographs and the group discussions) [22]. Finally, they met in a final meeting to: 1) get to know the other group members; 2) receive a personal portrait taken by the photographer; and 3) decide how they wanted their results to be used.

### Study design and participants

The research question that guided this study was: what were the experiences of the female participants involved in the Photovoice Villaverde project in terms of individual empowerment? In order to explore the meaning of this Photovoice experience, we used semi-structured interviews and participant observation as qualitative approaches.

We applied a purposive sampling strategy, inviting all the female individuals who had participated in the Photovoice Villaverde project to join the study. We decided to include only the female participants because of the multiple burden of inequality they face, often impeding their individual empowerment [23]. Public health practitioners (from the Public Health Institute of Madrid) undertook participants’ recruitment. They explained the study objective and invited them to participate. Finally, 10 women agreed to participate. Of the remaining 4 women, 2 did not respond, while the other 2 could not participate due to time constraints. Participants completed written consent forms, and gave permission to be put in contact to schedule an interview. Participants’ ages ranged from 36 to 60, two of them had a migrant background, and all of them lived in the area for at least 18 years and mostly with their husband and children. Table 1 displays participants’ sociodemographic characteristics.

**Table 1:** Sociodemographic characteristics of the participants (*n*=10), Madrid, Spain, 2016

Pseudonym	Age	Country of origin	Relationship status	Occupation	Highest level of education	Household monthly income
IP 1	36	Morocco	Married/ Partnered	Homemaker	Higher secondary	≥ 600€-1200€
IP 2	46	Spain	Married/ Partnered	Office cleaner	High-school graduate	> 1200€
IP 3	44	Spain	Widowed	Unemployed	Not a high-school graduate	< 600€
IP 4	44	Spain	Single	Retired	High-school graduate	≥ 600€-1200€
IP 5	46	Spain	Married/ Partnered	Homemaker	Not a high-school graduate	≥ 600€-1200€
IP 6	40	France	Married/ Partnered	Homemaker	University	> 1200€
IP 7	53	Spain	Married/ Partnered	Unemployed	Not a high-school graduate	> 1200€
IP 8	51	Spain	Married/ Partnered	Unemployed	Not a high-school graduate	≥ 600€-1200€
IP 9	60	Spain	Married/ Partnered	Unemployed	Not a high-school graduate	> 1200€
IP10	59	Spain	Married/ Partnered	Retired	Not a high-school graduate	> 1200€

### Data collection

One female researcher (KB), who did not participate in the original Photovoice project, with previous anthropological and qualitative research training, conducted all 10 semi-structured interviews. Interviews took place at different community facilities (e.g. local library). Interviews lasted between 20-35 minutes, were all audio-taped, and transcribed verbatim for analysis. We used open questions and narrative prompts to stimulate the interview partner’s account. These served as guidance, but also left spaces for the interview partner’s own ideas to come up during the interview. These characteristics allowed the interviewer to get access to the individual perceptions.

The topic guide (see Table 2) aimed at understanding the changes that Photovoice brought about along three initially outlined dimensions of empowerment. For instance, they related to how women experienced their participation, or what they felt they learnt from it. The topic guide remained flexible as emerging concepts were fed back to the participants in following interviews [24]. The topic guide did not include gender-specific questions, in order to avoid pointing their account towards one direction.

**Table 2:** Topic guide used in the study to guide participants’ interviews

How did you decide to take part in the Photovoice project?
ο What was your motivation for taking part?
ο Did you participate in other activities in your community before the Photovoice project (e.g. neighborhood associations...)?
Thinking back/Looking back on the Photovoice project, what experience did you have?
ο What were the activities or moments that you remember the most (and why)? (Examples: photography course, group debates, taking photos, meeting politicians…)
ο What did you learn from the other participants?
ο Do you think they had an influence on you and the way you see your neighborhood?
Thinking about the aim of the project of documenting with your camera everything that had to do with food in your neighborhood…How would you describe to someone else what you learned about your food environment, or what you learned about your neighborhood in this aspect?
ο Did it change the way you see your neighborhood?
ο Are there things, that you now know, but before weren’t aware of?
ο Were there topics that came up, that you did not have in mind?
From what you learned in the Photovoice project, can you give an example on how you apply/use this knowledge now?
ο Do you buy different products/ in different stores now?
ο Do you think projects like Photovoice can create further initiatives among the people who participate? Example?
ο Have you had any ideas for your own projects (individually or with others)?
ο Are you still active in the community (after Photovoice ended), what is your situation now?
ο Did you attend the meeting with the politicians? How did you like it?
ο Do you feel like there is something that does not allow you to apply and maintain what you have taken from the experience?
Did you know the other participants before or did you get to know them there?
ο Are you still keeping in touch?
ο When you went out to take the photos, did you talk to the people you took photos of?
ο Did they ask you what you were doing?
Let’s talk about your participation in the project… How did you feel? What did you learn about yourself in the project? What changed about you throughout the project?
ο Describe the experience of watching other people view your photos.
ο Did you do things that surprised you about yourself?

Researchers also attended public events where the Photovoice Villaverde project was showcased and where participants were present. These were either related to the dissemination of the project results, like exhibitions, or to public community meetings where the project results were discussed. Taking up the role of a participant observer [25], we created field notes of unstructured observations and conversations with the objective of shedding light on how the participants related to the project in public. These data enhance the validity of the findings by complementing and contrasting interview narratives with actual behaviour and expressions in social settings with less reactive dynamics; and by increasing reflexivity and rationality in the researcher-participant relationship [25].

### Data analysis

We conducted a direct qualitative content analysis [21]. Three previously outlined dimensions of empowerment provided guidance for the analysis of the results and thus represent a deductive, theory-driven approach.

One researcher analysed first all interview transcripts and observational field notes coding the data line-by-line (using the Nvivo software package), based on theory-derived categories and sub-categories [26]. To reduce the bias accompanying deductive-driven data analysis, we set out to approach the material probing the pre-defined categories while welcoming and integrating emerging themes that did not fit in the categories initially set out. This approach was informed by Neuman’s successive approximation [27] and Mayring’s qualitative content analysis [26]. We coded text that did not correspond to any of the established categories and created new categories where necessary. Two methods were used to increase the validity and reliability of the results [24]: The observational field notes were used to enrich data from the interview transcripts and to uncover contrasting perceptions. [28]. Then, a second researcher compared the codes with the corresponding textual material.

## Results

### Gain in knowledge and skills

Participants described that there were two central moments of knowledge creation during the Photovoice process: 1) the change of perspective while taking photographs; and 2) the collective production of knowledge during the small group discussions.

In relation to the ‘change of perspective while taking photographs’, participants emphasized that the camera lens allowed them to discover issues of their neighborhood: *“People who were looking for food, I maybe did not take them into account, and yes it is true that going outside with the camera and after having done the Photovoice I pay more attention and I see it more” (IP4)*.

The second central moment was the interaction between participants in the group sessions, where participants with different backgrounds and perspectives came together. The group sessions provided a space for knowledge exchange. Moreover, the photographs stimulated discussions in which they challenged their individual assumptions: *“(...) every one saw the same photo in a certain way, I mean, I saw an ordinary dish as something good to feed us all, and yet my partners did not (...) So, you learn from everyone, obviously, and it does not mean I was right, maybe they brought me round to their own terrain because we talked about it” (IP4).*

As an outcome of these two moments during the Photovoice process, the most salient theme mentioned related to becoming more conscious of their surroundings and being sensitizing to other perspectives. The latter related to the above-mentioned sharing of different perspectives and getting an insight into realities that are not one’s own: *“You interact with people of all social classes, and you learn (...) things that she does and I do not, so she does it out of necessity, and there are many people who would also do it if they knew” (IP5).*

### Change in self-perception

A change in self-perception was fostered by provision of a space where participants could express themselves and were valued for their opinion. All participants highly valued the fact of being recognized. Some related to the opportunity of voicing their opinion and being heard: *“That sentence: “giving us voice”, is the one I liked the most because it means there is someone who wants to hear your opinion, then, well, it forces you a little to reflect too” (IP6).*

This was also perceived as a result of the way of working within the Photovoice process, as the facilitators treated them as peers and did not steer participants into one direction but rather “*left the field open for [them] to do what [they] wanted*” (IP1). This equal power relation was also underpinned by the frequent use of the word “*collaborate*” when referring to the relationship between participants and university-based researchers in the Photovoice project.

One of the perceptions most predominantly voiced by the participants related to being perceived in a different way, being acknowledged for their work among a variety of actors (from friends and family to the research partners, local decision makers and the wider public). Participants, for instance, expressed that researchers valued and gave worth to their ideas within the project: *“To be that person in whom they have trusted, in whom they have believed, right? You have value for the project” (IP 1)*

The aspect of being recognized and acknowledged became apparent at the dissemination activities (e.g. community meetings, photographic exhibitions), where participants presented their project results, and were asked about their experience by other residents. One woman mentioned how walking through the streets she is sometimes recognized as being part of the project. Finally, the participants also entered the field of producing and sharing local policy recommendations, where they turned into visible actors, something that none of them anticipated, as IP 1 explains: *“We did not have such a deep-rooted concept that we are going to do something so great, (...) on top of it this is going to be done in Cibeles* [the most important and recognizable Cultural Centre in Madrid]*, for example, with the mayor of Madrid ...” (IP 1)*

For many women, the involvement in the project added perceived self-worth, as for example, they felt like occupying a more active role and being able to help others. Fostered by a substantial weight loss during the project, one woman completely transformed her self-image, something that also became apparent at the events where the project was disseminated: She visibly enjoys being in pictures and to appear in public*: “I was fat as a cow, and look how I am now! I have changed overnight, now people hear me speaking and say Jesus! I am another person, I mean, I like myself more, I love myself more...”(IP 3)*

### Access to and use of resources

The participants’ narratives showed that the social relations that they established were one of the main resources that arose from the Photovoice experience. They extended their social network by building new links with different actors. Thus, they got access to people with different sets of knowledge and learned from them. On the one hand, these could be co-participants with different social backgrounds, on the other hand these could be people outside of the project, where Photovoice linked participants to different local actors they could benefit from (e.g. nutrition experts).

**Fig. 1:**
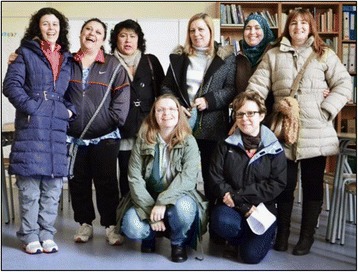
Female participants of the Photovoice project conducted in the District of Villaverde, Madrid (Spain), 2016

One of the main positive personal outcomes of their participation was the friendship they built or strengthened with the other group members (see Fig. 1). They described how they were now “*inseparable*” (IP 1) from some of the participants, they would have never got to know otherwise, and how these relations could also be a source of support and security, and that could be completely trusted “*for anything you need*” (IP1), for example, if one of their children had problems.

Furthermore, the participants entered fields of social interaction that are normally hard to access for residents of the low-income district of Villaverde. This relates to building long-term relationships with public health practitioners of the local health promotion center and local decision makers as for example offering to have a “*meeting with them again*” (IP 10).

Engagement in the Photovoice project stimulated local actions in different ways, serving as a trigger for initiatives: Two women joined the parents’ association of the local high school as a means to keep their active role in the community; while four of the women put together a local radio program, as *“a continuity of Photovoice” (*IP1*),* where they talk about the neighborhood issues and present their perspective on local topics. Members of the newly founded radio program took advantage of the publicity of the Photovoice project as a resource to promote their own initiative: *“(…) so, if we are going to the event [Cibeles exhibition] and we can make it happen to meet with the mayor (…) we can get an interview with her and make a fantastic impression…” (IP 6).* IP 6 also had the idea of collecting participants’ recipes and all together publish a cookbook, also to show that they too had *“that ability to take initiative, to say ‘apart from this, we create another project (…)”* (IP6).

**Fig. 2:**
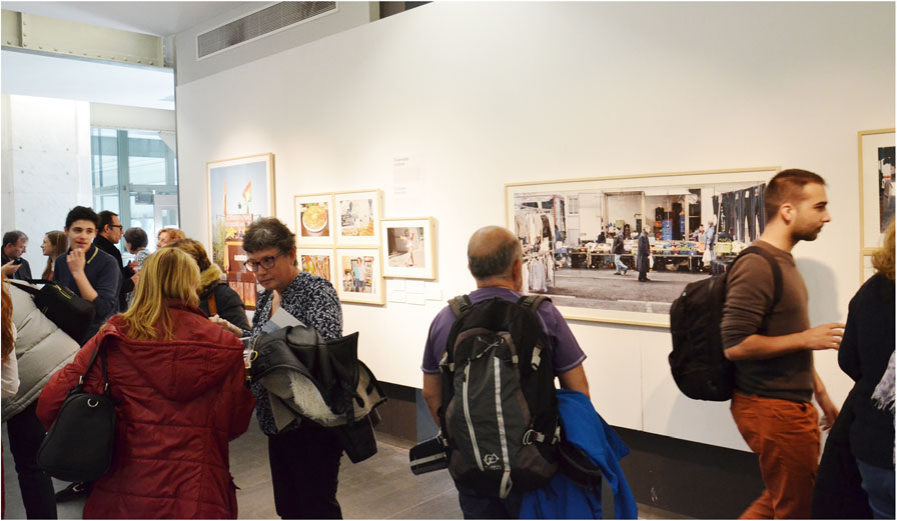
Public presentation of the photographic exhibition of the Photovoice project, Madrid, 2017. Participants chose the final photographs they wanted to be included in the photographic exhibition and were involved in all dissemination activities

The Photovoice project received public attention in the local sections of three national newspapers and was featured on several radio programs. One participant was interviewed in the largest audience weekend radio program in Spain. Furthermore, participants selected the photographs they wanted to include in the photographic exhibition of the project, which has been shown in four different settings in Madrid (see Fig. 2), and also in the European Parliament, with them participating. One of the interviewee’s Photovoice pictures was printed in the largest newspaper in Spain.

These moments yielded the possibility for the women to keep engaged and position themselves in public. We organized two different community meetings, where Photovoice participants presented and discussed with researchers and local policy-makers their study results, in order to translate them into concrete initiatives to improve the neighborhood food environment. Others used these public events as an opportunity to advocate for their causes, from raising awareness about scarce resources and poverty in Villaverde to suggesting new ways of administrating the food banks. We also edited a free-downloadable photobook (https://hhhproject.eu/hhh-sub-studies/photovoice/photovoice-publications/), a video (https://www.youtube.com/watch?v=VIiFggKzVas), and a website (www.hhhproject.eu/photovoice), which were shown in all dissemination meetings.

## Discussion

We explored and described the individual experiences of empowerment of the female participants who had engaged in the Photovoice Villaverde project, in terms of knowledge gain, self-perception changes and access to resources. Changes occurred in all these three dimensions reflecting the positive experiences of female individuals involved in the Photovoice project.

Among the findings that emerged from this study, participants described a personal growth (acquiring new knowledge and developing critical awareness). The participation process in the Photovoice project provided the ground for participants to develop a more critical, attentive and empathetic view on their neighbourhoods and their local environment. Interestingly, this new knowledge was not specifically expanded in terms of the local food environment. Women rather became more critical, attentive and empathetic with their community [29]. Just as Foster-Fishman et al. note, “these insights did not replace the participants’ own perspectives, but instead broadened them.” [30]). As Teti et al. also found, Photovoice methodology stuck out as an important driver for empowerment as an outcome [16]. The use of the camera also proved to be a transformative tool by itself as it has the potential to stimulate a process of reflection among the participants. Previous studies using Photovoice also found that participants became more empowered and socially conscious about their environment [22, 31].

As described by Freire, participating in the Photovoice project clearly initiated a process of critical reflection and “conscientização” [4] for many of the women as they used it as a tool to challenge their own assumptions. Considering the idea that participating in the community is seen as an indicator for empowerment, the women were all already empowered in a certain way, however the levels and characteristics of their community engagement were contrasting. We consider it most useful to see social participation as “the backbone of empowering strategies” that may still lead to very different outcomes for the participants [32].

Our findings also show the active role that participants had in the Photovoice project: they created knowledge, became co-researchers themselves, advocated for their ideas – stipulating participation as a central aspect of Photovoice. This draws on the notion that residents are the actual experts on their environment, and the ones who should guide the actions needed to foster policy changes at the community-level [19, 31, 33]. In participatory action research (PAR), the end goal is to involve participants during the entire research project [12]. In this context, our participants were involved in all dissemination and outreach activities of the project. Moreover, they collected and analysed all data of the research project, following a participatory data analysis. Participants have also collaborated, as co-authors, in the elaboration of the research articles of the project [18]. According to previous studies, it is critical that people affected by a project stay involved in order to determine its success, particularly critical in an empowerment project [8].

In this line, the context and the group dynamics between all involved actors were crucial for their positive outcomes [34]. Our decision to conduct separate Photovoice groups by gender was based on recognizing the evidence of disparities in health between men and women [23]. By doing so, it allowed us to capture gender differences, and gave women the opportunity to express their opinions in an environment free from the power pressure of men [35]. An internal contextual influence that might be difficult to recreate is the highly positive connotation the participants gave to the relationships in the group that nurtured the individuals’ emotional engagement and their positive self-perception and substantially shaped the strong group dynamics [15]. An important local contextual factor could be the existing tradition of social participation and the residents’ identification with their neighbourhood in Villaverde, a historically underserved area. Madrid is a city where residents identified a lot with their neighbourhoods. In this context, it can hence pose a challenge to predict empowerment outcomes for a similar project in a different community setting with potentially less established group dynamics – an issue widely encountered in community-based participatory research (CBPR) [32].

The Photovoice project created a space for social interaction from which friendships and additions to the individuals’ social network arose. These new relationships represent the most highlighted positive outcome from the participants’ perspective. Such positive social interactions and relations are widely seen as one of the key elements of empowerment [32, 36, 37]. They can be characterized by its social cohesion, the “extent of connectedness and solidarity among groups in a society” [38] measured by levels of social trust, reciprocity and the existence of institutions that create the basis for positive social interaction (ibid.).

Some of the participants in Villaverde extended their roles as social actors in the community. They powerfully used the increased self-confidence they received from the recognition received in the project and the newly established social contacts as a resource for action. This can most apparently be observed through the local radio program several of the participants started. It draws on the Photovoice experience, as they use a documentary tool, in this case the audio recorder, to engage with and reflect on their neighbourhood.

The present study was subject to several limitations. Specifically, three aspects arouse that might be restraining Photovoice to develop its full potential as a tool to gain empowerment. Firstly, Photovoice participation is an intensive commitment of time with weekly meetings over months. So, participants have to embed their engagement into their lives and negotiate between competing interests, responsibilities and roles that may restrain their participation. The one most salient external limitation for the women was grounded in existing gender imbalances. Many women pointed out that their obligations and responsibilities in their families limited their engagement in the project. Thereby, Photovoice projects in general are likely to introduce a selection bias. In this study, 4 of the initial female participants (n=14) could not be interviewed, they might have been less engaged with the project and it would therefore have been valuable to capture their perspective. With the intention to compensate this missing information a biographical component was included in the interviews. Secondly, the two female groups were residents of two different and socially distinct neighbourhoods within the District of Villaverde and did not seem to create a common vision of what they wanted to act on. Regardless of the lack of this common ground, both groups built bonds and learnt from each other [11]. Thirdly, as in other Photovoice projects, this process was not always built on established structures allowing participants to keep engaged or act on social transformation in the most efficient and meaningful way [39]. As Wang pointed out, the methodology itself may have “stopped short of engaging participants in conceptualizing and participating in action steps needed to address their needs.” [11]. In their narratives, participants frequently mentioned the possible transient character of the research project. The original phase of organized group interaction had an “expiration” date. Nevertheless, the project has evolved and continued over the years and participants are still engaged as of today (January 2108) with the research team as they decided to translate their research results into a set of concrete strategies to improve their local food environment. As such, they have met with several policy-makers from the City Council, to present and discuss with them the set of policy recommendations that they produced. Thereby, researchers, local health practitioners (from the local health promotion centre in Villaverde) and participants are still committed to this project, and have established a structure to work hand in hand with local institutions, which further implicated participants after the Photovoice finished.

### Public health implications

Our *Photovoice Villaverde* project was originally designed as a participatory research project to understand the local food environment in an underserved and low-income area of Madrid. Being a qualitative study, we should be careful generalizing project results to other individuals. However, the experiences regarding individual empowerment described by female participants makes it likely that other participants in other Photovoice projects may also experience empowerment changes.

Photovoice research teams need to work as closely as possible to the local structures and institutions to facilitate meaningful long-term participation. However, goals and strategies and limitations for such social change have to be clearly and realistically defined and communicated [40]. This is very important to avoid “*raising false hopes or unrealistic expectations [for policy change] amongst the participants of Photovoice projects*” [39].

## Conclusions

Female participants in a Photovoice research project that took place in an underserved urban community showed positive individual empowerment results. Women acquired new knowledge developing critical awareness of their community, received social recognition and expanded their social networks.

## Abbreviations

CBPR: Community based participatory research
PAR: Participatory action research
